# Modeling Human Impact on the Distribution of the Only Existing Species of *Euryale*

**DOI:** 10.1002/ece3.73994

**Published:** 2026-07-08

**Authors:** Yanxuan Li, Ningjing Zhang, Zhongyi Gao, Jiani Shi, Bingru Fang, Jishu Guo, Peng Xie, Yun Zhang, Wen Xiong, Kun Xu

**Affiliations:** ^1^ Hubei Key Laboratory of Edible Wild Plants Conservation and Utilization, College of Life Sciences Hubei Normal University Huangshi China

**Keywords:** anthropogenic impact, conservation, *Euryale ferox*, global change, species distribution model

## Abstract

Human activity has a profound impact on the distribution of a number of species, especially those with ecological and economic importance. *Euryale ferox* Salisb., an annual macrophyte native to South, Southeast, and East Asia, has long been cultivated for its fruits and seeds in north India and east China. However, classified as endangered in Japan, South Korea, Kashmir Himalaya, and Taiwan, China, *E. ferox* is the relict species of the genus that has been under both natural and human disturbances. Under rapid global change, how human activity affects the distribution of *E. ferox* in Asia remains unknown. To answer whether humans promote or limit its distribution, we compile by far the most complete set of 404 records of occurrence of *E. ferox* from the Russian Far East and 10 countries in Asia, and build species distribution models using machine learning methods. The results show that temperature of the coldest month is negatively associated with the occurrence of *E. ferox* , while the positive association between it and anthropogenic nitrogen deposition is more important for explaining its distribution. Its distribution is projected to decline by 8.3%–16.7% under four climate change scenarios in the 2050s if the levels of human activity remain unchanged. Given the genetic, ecological, medicinal, and economic importance of *E. ferox* , it is necessary to conserve its wild populations under global change.

## Graphical abstract

Temperature of the coldest month is negatively associated with the occurrence of *Euryale ferox* while the positive association between it and anthropogenic nitrogen deposition is more important for explaining its distribution. Its distribution is projected to decline by 8.3%–16.7% under four climate change scenarios in the 2050s if the levels of human activity remain unchanged. Given the genetic, ecological, medicinal, and economic importance of *E. ferox* , it is necessary to conserve its wild populations under global change.

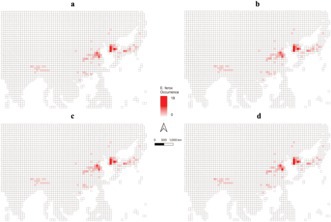

## 1 Introduction

*Euryale ferox* Salisb. (Nymphaeaceae), known as prickly waterlily or makhana, is an annual rooted floating macrophyte native to Asia, distributing in a variety of freshwater ecosystems from Pakistan to Japan (Löhne et al. 2008; Kim et al. 2010; Imanishi et al. 2011; Kumar et al. 2016). At the early growth period, its leaves rapidly extend from underwater to occupy a large area of water surface, an adaptive feature for the aquatic environment (Yang et al. 2020; Wu et al. 2022). Like many macrophytes, *E. ferox* accumulates toxic metals from water, purifies water, and stabilizes the aquatic environment (Rai et al. 2002; Ni et al. 2021; Wang et al. 2022). It is also important for conserving the biodiversity of its habitats by providing food for wintering water birds (Zhou et al. 2020) and shelters for aquatic insects (Guru et al. 2024). Although it is the only existing species of the genus *Euryale* (Huang et al. 2015), genetic diversity is detected among its wild populations in India, Japan, and China (Quan et al. 2009; Imanishi et al. 2011; Kumar et al. 2016). Despite it being much unknown for the evolutional, developmental, and reproductive biology of *E. ferox* (Kadono and Schneider 1987; Huang et al. 2015; Liu et al. 2018; Yang et al. 2020), there is a long history of utilization for its ecological, nutritional, and pharmaceutical values in Asia, especially in China and India (Jha et al. 1991; Das et al. 2006; Kapoor et al. 2022; Manish 2022; Jiang et al. 2023). Specifically, *E. ferox* has been a folk medicine for over five thousand years in China, widely used in Chinese Traditional Medicine for its antioxidant activity and therapeutic effect (Lee et al. 2002; Jiang et al. 2023). It has also been used as a medicinal plant in India, Nepal, and Myanmar for hundreds of years (Jha et al. 1991; Manish 2022). Recently, the nutritional values of its seeds are intensively utilized in North Bihar, India (Kapoor et al. 2022; Farooqi et al. 2025) and the Yangtze River Delta, China (Zhou et al. 2020; Zhang et al. 2022).

Although *E. ferox* has recently been widely cultivated as a crop plant for fruits, that is, fox nut, gorgon nut, or qian shi, in north India and east China (Devi and Deka 2022; Zhang et al. 2022), the distribution of its wild populations shrank in east India, northwest China, South Korea, Japan, and Southeast Asia over the past century (Jha et al. 1991; Li et al. 2006; Imanishi and Imanishi 2014; Kim et al. 2010, 2018). In fact, it is listed as an endangered species in Japan, South Korea, Kashmir Himalaya, and Taiwan, China due to habitat loss, environmental pollution, and global warming (Rai et al. 2002; Imanishi and Imanishi 2014; Kim et al. 2018; Semwal et al. 2021; Manish 2022; Lee and Liu 2026). For example, *E. ferox* is classified as a vulnerable vascular plant protected by the national law in South Korea under the threat of interspecific competition and environmental pollution (National Institute of Biological Resources 2014). In addition, diseases and pests of *E. ferox* become more frequent in its cropland than before, which further threaten conservation for this species (Srivastava et al. 2022; Padala et al. 2023; Guru et al. 2024). Efforts have been made by different countries to protect *E. ferox* from the risks of population decline, genetic diversity loss, and even local extinction (Jha et al. 1991; Imanishi and Imanishi 2014; Kim et al. 2018; Liu et al. 2018; Semwal et al. 2021; Kashyap and Shukla 2024). Specifically, the efforts on predicting its suitable habitats based on bioclimatic variables using species distribution models are informative for conservation planning (Kim et al. 2018; Semwal et al. 2021). The existing bioclimatic model estimates that the probability of occurrence of *E. ferox* in South Korea is positively associated with mean annual temperature and moisture index while is negatively associated with elevation, slope, and distance to the nearest river (Kim et al. 2018). This model also indicates that it is more likely for the species to occur at the habitats with intermediate temperature seasonality, annual precipitation, and flood area than at the other areas (Kim et al. 2018). In India, it is estimated that the range of highly suitable habitats for *E. ferox* may remain stable by 2050 but may shrink by 2070 under climate change according to the national species distribution model (Semwal et al. 2021).

Based on the projections by the two existing species distribution models, *E. ferox* is under the threat of local extinction in east India and South Korea (Kim et al. 2018; Semwal et al. 2021), evidenced by the decadal decline of its wild populations in Asia (Kadono and Schneider 1987; Jha et al. 1991; Imanishi and Imanishi 2014; Kim et al. 2018). However, it remains uncertain about the potential distribution of *E. ferox* under global change, especially in Southeast Asia, China, and Japan. Other than climate, the impact of human activity, such as land use change, anthropogenic nitrogen deposition, and human population growth (Liu et al. 2013; Han et al. 2019; Tan et al. 2025; Rao et al. 2026), should also be considered as potential factors affecting the distribution of *E. ferox* . What roles do humans play in the distribution and survival of this endangered species? Considering the ecological and economic importance of *E. ferox* , it is necessary to develop a continental species distribution model based on its current occurrence in Asia to estimate the impact of climate and human activity on its distribution and to project its future distribution under global change. This study compiles the most complete recent records of occurrence of *E. ferox* from the Russian Far East and 10 countries in Asia, and applies machine learning methods to project the occurrence of the species in Asia under four global change scenarios. The results will inform sustainable utilization, cultivation, and conservation of *E. ferox* , especially its wild populations, in Asia.

## 2 Methods

### 2.1 Occurrence Records

**FIGURE 1:**
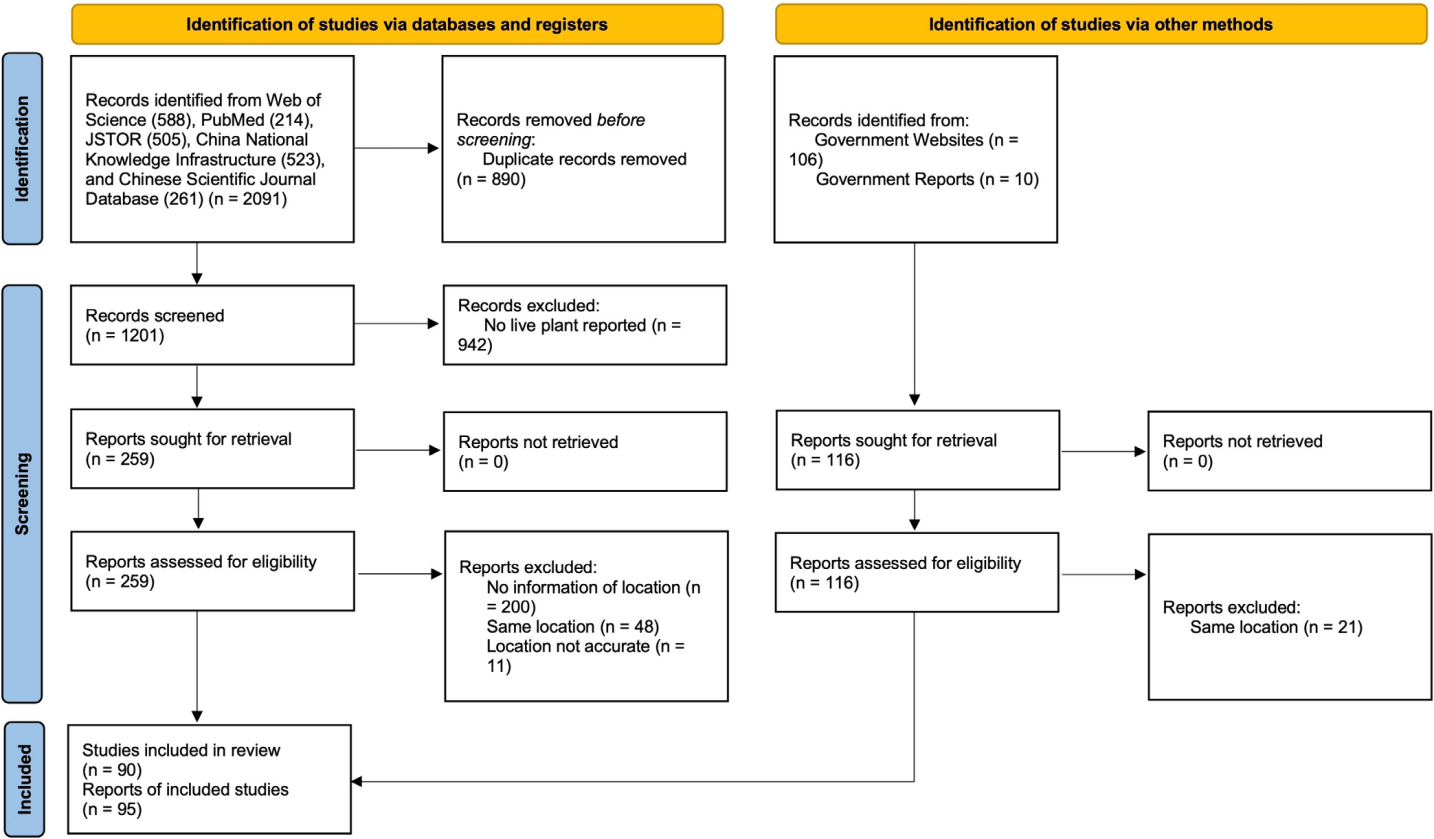
The PRISMA 2020 scheme for compiling the 404 occurrence records of *E. ferox* in Asia from studies and reports.

We compiled the records of occurrence of *E. ferox* by conducting a literature search following the PRISMA 2020 checklist (Page et al. 2021). The keywords used for the search from Web of Science, PubMed, and JSTOR were the Latin and common names of the species, namely “ *Euryale ferox* ” OR “ *E. ferox* ” OR “foxnut” OR “fox nut” OR “makhana” OR “Gorgon plant” OR “Gorgon nut” OR “qianshi” OR “qian shi” OR “prickly waterlily”. We also searched the China National Knowledge Infrastructure and the Chinese Scientific Journal Database as well as government reports and news with the keywords in Chinese to enhance the coverage of the records in China (Figure 1). Only the results with information about the locations of the populations of the species growing in water bodies were included, and the records for those grown under laboratory conditions were excluded. Although it was clear whether the populations were cultivated or not, it was not possible to determine if there were both wild and cultivated populations at the same location for every record (Jha et al. 1991). Instead, the pooled occurrence records were useful for analyzing the impact of human activity on its distribution (Tan et al. 2025; Li et al. 2026). To ensure that the compiled records represent the current distribution of *E. ferox* in Asia, we excluded the occurrence records only reported before 2005. For instance, the species went extinct in the Tarim Basin, Xinjiang Uygur Autonomous Region (Li et al. 2006) and Sun Moon Lake, Nantou County, China (Lee and Liu 2026) and Dal Lake, Kashmir Himalaya (Jha et al. 1991; Mir et al. 2020) due to human disturbances in the mid to late 20th century, which might be reported in the past literature but not in the recent articles. Including these past records of the extinct populations would increase the risk of overestimation for the current distribution of *E. ferox* in Asia. We also checked and removed duplicated records at the same locations (< 5 km) from multiple sources of information as well as the records without information of accurate locations. The coordinates of each record of occurrence were obtained from Google Earth based on the description of the location if there was no coordinate available.

We then divided the study area covering its current distribution (5° N–55° N, 70° E–145° E) into 3750 1‐degree grids to handle the issue of overrepresentation of the records at the local scale (Tan et al. 2025). We excluded 1117 grids completely in the sea by intersecting the study area with the global terrestrial boundary (Runfola et al. 2020). In each of the remaining 2633 grids, the number of occurrences of *E. ferox* was counted. We then calculated the global Moran's *I* (Moran 1950) of the number of occurrences per grid among the 125 grids with at least one occurrence record to assess the spatial autocorrelation in Asia. Hot and cold spots of its occurrence were analyzed using the Getis‐Ord Gi* (Getis and Ord 1992). The 1‐degree spatial resolution was appropriate for modeling its distribution in the study area as the ratio between the number of grids with at least one occurrence record and the number of those without any record was 5.0% (Evans et al. 2011; Mushagalusa et al. 2022; Tan et al. 2025).

### 2.2 Explanatory Variables

In total, we compiled 35 variables of climate (14 variables), habitat condition (11 variables), and human activity (10 variables) as the candidate explanatory variables for predicting the occurrence of *E. ferox* in Asia. First, we extracted grid mean of 12 bioclimatic variables for the 2633 grids, including mean annual temperature (tavg; °C), temperature of the coldest month (tcm; °C), temperature of the warmest month (twm; °C), mean annual precipitation (ptot; mm), precipitation in summer (psumm; mm), in winter (pwint; mm), in the driest month (pdm; mm), in the driest month in summer (pdsumm; mm), in the driest month in winter (pdwint; mm), in the wettest month (pwm; mm), in the wettest month in summer (pwmsumm; mm), and in the wettest month in winter (pwmwint; mm), from the 1‐km 1984–2013 climate normal dataset (Cui et al. 2021). These data represent a more recent and more accurate climate normal for Asia than the 1970–2000 climate normal from the WorldClim dataset (Cui et al. 2021). We also extracted two hydroclimatic variables, namely mean annual potential evapotranspiration (et0; mm) and mean aridity index (ai), from the 30 arc‐seconds global hydroclimatic dataset (Zomer et al. 2022). Though the time periods of annual potential evapotranspiration and aridity index data, that is, 1970–2000, differ from those of the 12 bioclimatic variables, the trends in annual potential evapotranspiration and aridity index are stable in Asia, suggesting that these two hydroclimatic data are comparable to those in 1984–2013 (Huo et al. 2013).

Second, in terms of habitat conditions, we extracted data for vegetation, topography, hydrography, and soil conditions for the grids. We extracted the mean annual Normalized Difference Vegetation Index (NDVI) in 2015 from the 5‐arcmin GIMMS NDVI dataset to represent the vegetation conditions of the grids (Li et al. 2023). The decadal change in NDVI from 2005 to 2015 (NDVIc) indicating the change in habitat condition was also extracted from the same dataset. In addition, we calculated the mean elevation (elev; m), slope (slope; °), and terrain ruggedness index (TRI) of the grids from the 30‐arcsec global Digital Elevation Model (DEM) (Hastings et al. 2000). These topographic variables were found associated with plant species distribution (Rao et al. 2026; Tang et al. 2026). We also extracted the upstream area (hydacc; ha) and upstream flow length (hydlup; m) of the rivers within each grid from the 30 arcsec global hydrography data (Lehner et al. 2008) to represent the hydrographic conditions of the grids. In terms of soil conditions, the average soil and sedimentary deposit thickness (thickness; m) (Pelletier et al. 2016), and organic carbon relative to topsoil weight in percentage (toc), pH in H_2_O (tpH), and cation exchange capacity (tCEC; cmol kg^−1^) of topsoil (0–30 cm of the soil layer) (FAO and IIASA 2023) were extracted for the grids from two 30‐arcsec global soil databases (Pelletier et al. 2016; FAO and IIASA 2023). These 11 variables represent the habitat conditions, including vegetation, terrain, hydrography, and soil conditions, of the grids.

Last, in terms of human activity, we extracted the majority of land cover type (LUmajor) in 2015 from the 5‐km land cover dataset (Liu et al. 2020), mean nitrogen deposition as NH_x_ (nhx; gN) and as NO_y_ (noy; gN) in 2015 from the 5‐arcmin anthropogenic nitrogen deposition from atmosphere to surface dataset (Tian et al. 2022), mean gross domestic production (GDP; USD) in 2015 from the 5‐arcmin annual GDP dataset (Kummu et al. 2025), and mean human population density (pop; km^−2^) in 2015 from the 30‐arcsec global human population dataset (Doxsey‐Whitfield et al. 2015). In addition, the decadal changes in the percentage of built‐up land (LULC) (Ning et al. 2018), mean decadal changes in NH_x_ (nhxc), NO_y_ (noyc), GDP (GDPc), and human population density (popc) from 2005 to 2015 of the grids were calculated to reflect the intensity of recent human activity (Jiang et al. 2025; Tan et al. 2025). The spatial resolutions of the human activity data between 1 and 5 km were sufficient to account for their variation among the 1‐degree grids (Xu et al. 2025). In total, there were 14 variables of climate, 11 of habitat condition, and 10 of human activity, of which NDVI and five variables of human activity were the decadal changes. The spatial correlation between each pair of these 35 variables was examined using the modified *t*‐test (Dutilleul et al. 1993). We excluded 22 variables with the absolute values of Pearson correlation coefficients (|*r*|) above 0.7 to handle spatial correlation issues (Tan et al. 2025) before building the species distribution models with the remaining 13 variables, namely pdm, tcm, ai, NDVIc, elev, hydlup, thickness, toc, tpH, LUmajor, LULC, noy, and GDP (Figure S1).

### 2.3 Species Distribution Models

Given the relatively small number of grids with records of occurrence of *E. ferox* (*n* = 125), we used the random forest regression method (Breiman 2001), a machine learning technique widely used for predicting species distribution (Evans et al. 2011; Valavi et al. 2021), to estimate the number of occurrence per grid in Asia. As the random forest model was a non‐parametric ensemble‐based prediction model, using gridded population density data, as in this study, would decrease the sensitivity of the model to spatial autocorrelation in occurrence and increase the spatial representation of the prediction for the actual populations (Sinha et al. 2019). When building the models with different sets of tuning parameters (mtry from 1 to 13 and mtree from 100, 200, 300 to 1000), we conducted the 10‐fold cross‐validation, that is, 10% of the grids with and without occurrence records of *E. ferox* as the testing set, to compare the performance metrices of models on predicting the number of occurrence per grid for the testing set. The four performance metrices were mean absolute error (MAE), rooted mean squared error (RMSE), relative absolute error (RAE), and root relative squared error (RRSE) (Sokolova et al. 2006). We then estimated the model with the optimal set of tuning parameters and the full set of data, and evaluated the importance of each of the 13 explanatory variables to the model as percentage increase in mean square error (%IncMSE) and decrease in node purity (IncNodePurity) when removing that variable from the model (Breiman 2001). The partial dependence of the number of occurrence on each of these variables is estimated as the marginal effect of this variable (Greenwell 2017).

### 2.4 Projections

We fitted the projected climate normal under four emissions scenarios (RCP 2.6, 4.5, 6.0, and 8.5) in the 2050s (Cui et al. 2021) to the parameterized random forest regression model to project the distribution of *E. ferox* in Asia given the other variables unchanged. The four emissions scenarios adopt four forcing levels of 2.6, 4.5, 6.0, and 8.5 W/m^2^ by the end of the 21st century (Riahi et al. 2011), with the RCP 2.6 as the mild, 4.5 and 6.0 as the realistic, and 8.5 as the intensive emission scenarios (Thomson et al. 2011). The difference between the current and the projected numbers of occurrence of the species was calculated, and the 1st and 99th percentiles of such difference were used to evaluate the change of its distribution under climate change (Xu et al. 2025).

## 3 Results

### 3.1 Current Distribution

**FIGURE 2:**
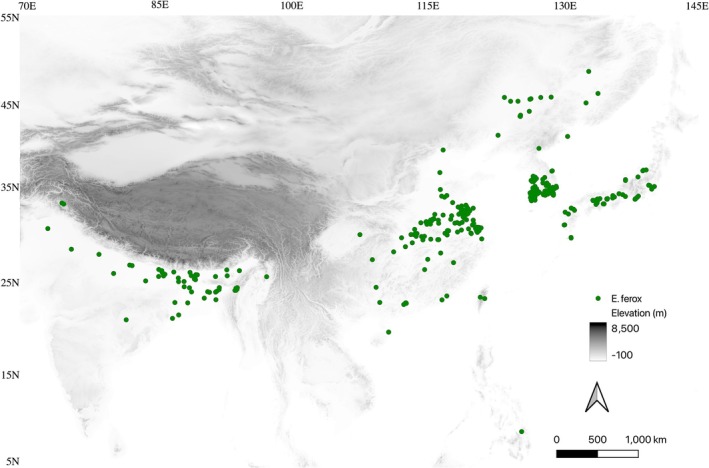
Current distribution of the 404 records of occurrence of *E. ferox* across 10 countries in Asia as well as the Russian Far East. The study area spans from 5° N, 70° E to 55° N, 145° E with elevation (m) as the base map.

**FIGURE 3:**
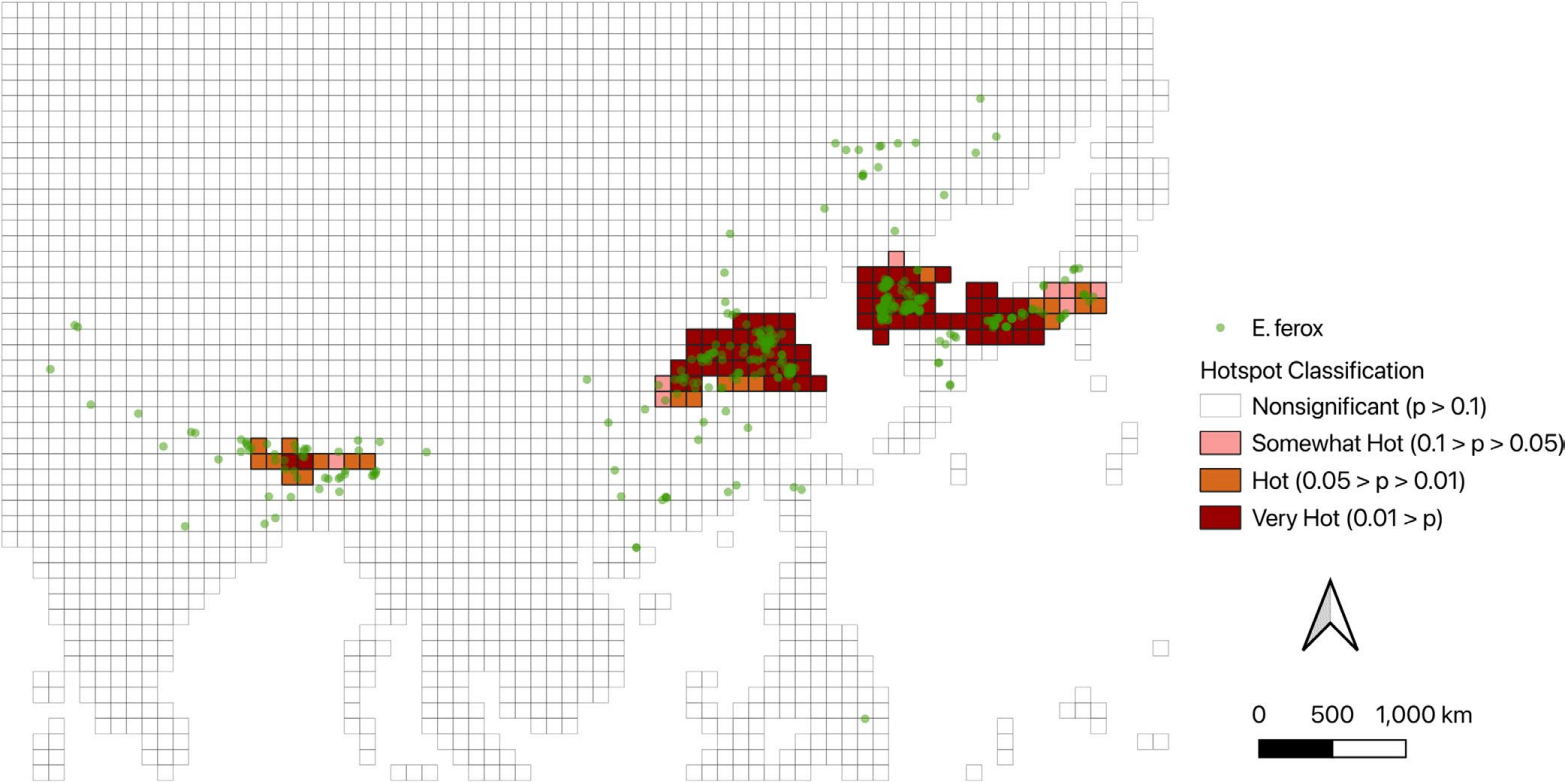
Hotspots of the occurrence of *E. ferox* in the gridded study area. The 404 records of occurrence are the green dots. No coldspot but only hotspots are detected based on the *z*‐scores and *p*‐values from Getis‐Ord Gi*. The three classes of hotspots are determined by the *p*‐value of the Getis‐Ord Gi* of each grid as somewhat hot (pink), hot (orange), and very hot (red).

In total, 404 records of occurrence of *E. ferox* from the Russian Far East and 10 countries in Asia are compiled (Figure 2). These records range from 9.0° N to 48.8° N and from 73.1° E to 140.2° E, matching the known distribution of the species in Asia (Löhne et al. 2008). According to the records, it is widely cultivated in India and China, frequently observed in South Korea and Japan, and occasionally observed in Pakistan, Nepal, Bangladesh, Myanmar, Philippine, North Korea, and the Russian Far East (Figure 2). The Global Moran's *I* for the number of occurrences of *E. ferox* is 0.103 (*p*‐value = 1.55 × 10^−15^), indicating a positive spatial clustering of its distribution (Moran 1950). The hotspot analysis of its occurrence based on the Getis‐Ord Gi* shows that the species strongly aggregates in north India, east China, and South Korea, whereas no coldspot is present (Figure 3).

### 3.2 Species Distribution Model

**FIGURE 4:**
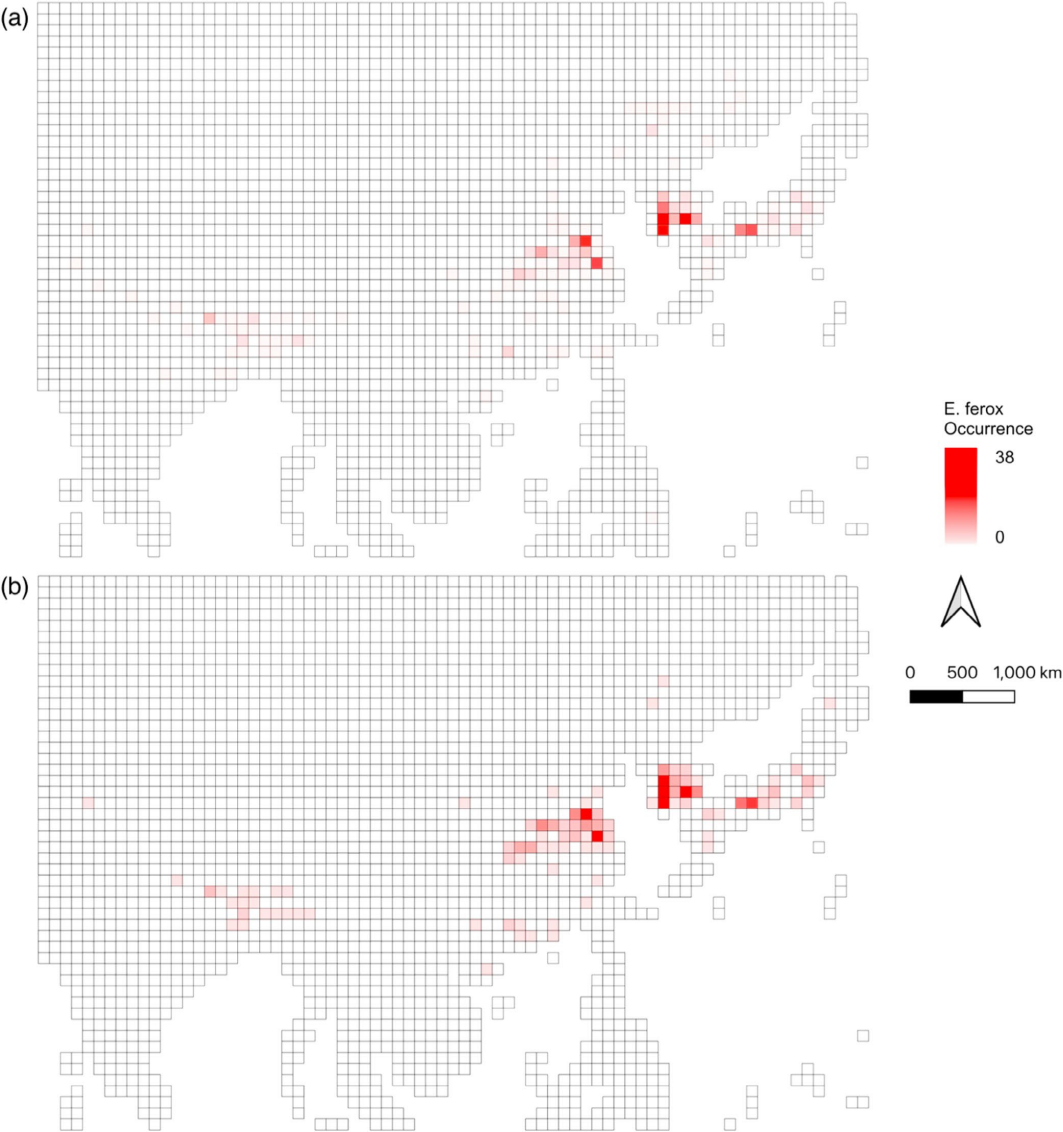
Distribution of observed (a) and predicted (b) number of occurrence of *E. ferox* per grid in the study area. The white‐to‐red gradient illustrates the number of occurrence per grid ranging from 0 to 38.

The optimized model is tuned with the values of the mtree and mtry parameters as 500 and 4, respectively, which explains 20.01% of variance in the number of occurrences of the species per grid. The MAE, RMSE, RAE, and RRSE of the predicted number of occurrences by the fitted model are 0.272, 1.383, 0.739, and 0.894, respectively. By comparing the observed (Figure 4a) and predicted (Figure 4b) numbers of occurrence per grid, we identify that all three clusters of hotspots in North India, east China, and South Korea (Figure 3) are captured by the model.

**FIGURE 5:**
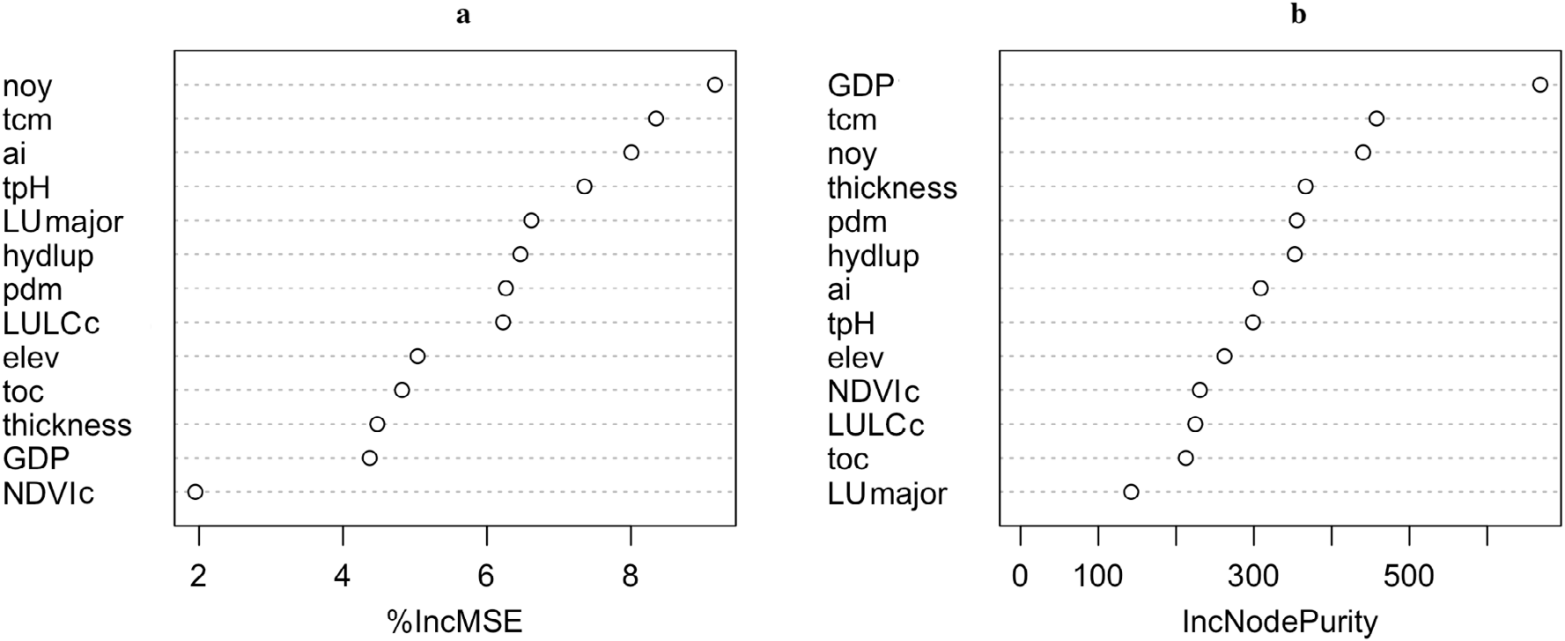
Variance importance measured by %IncMSE (a) and by IncNodePurity (b) of the 13 explanatory variables in the fitted random forest regression model for the number of occurrence of *E. ferox* .

**FIGURE 6:**
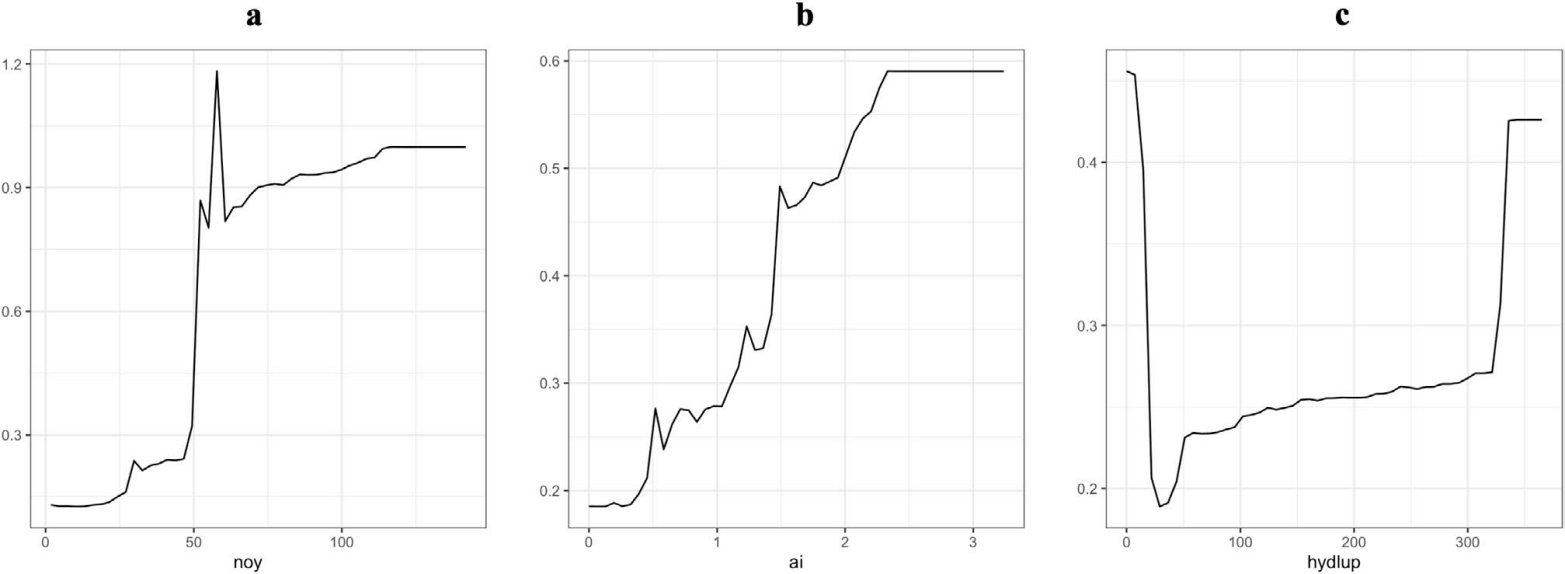
Partial dependence of the number of occurrences of *E. ferox* on nitrogen deposition as NO_y_ (noy) (a), aridity index (ai) (b), and upstream flow length of the rivers (hydlup) (c) based on the random forest regression model. The *y* axis is the estimated number of occurrences per grid by marginalizing over the effects of the other variables.

**FIGURE 7:**
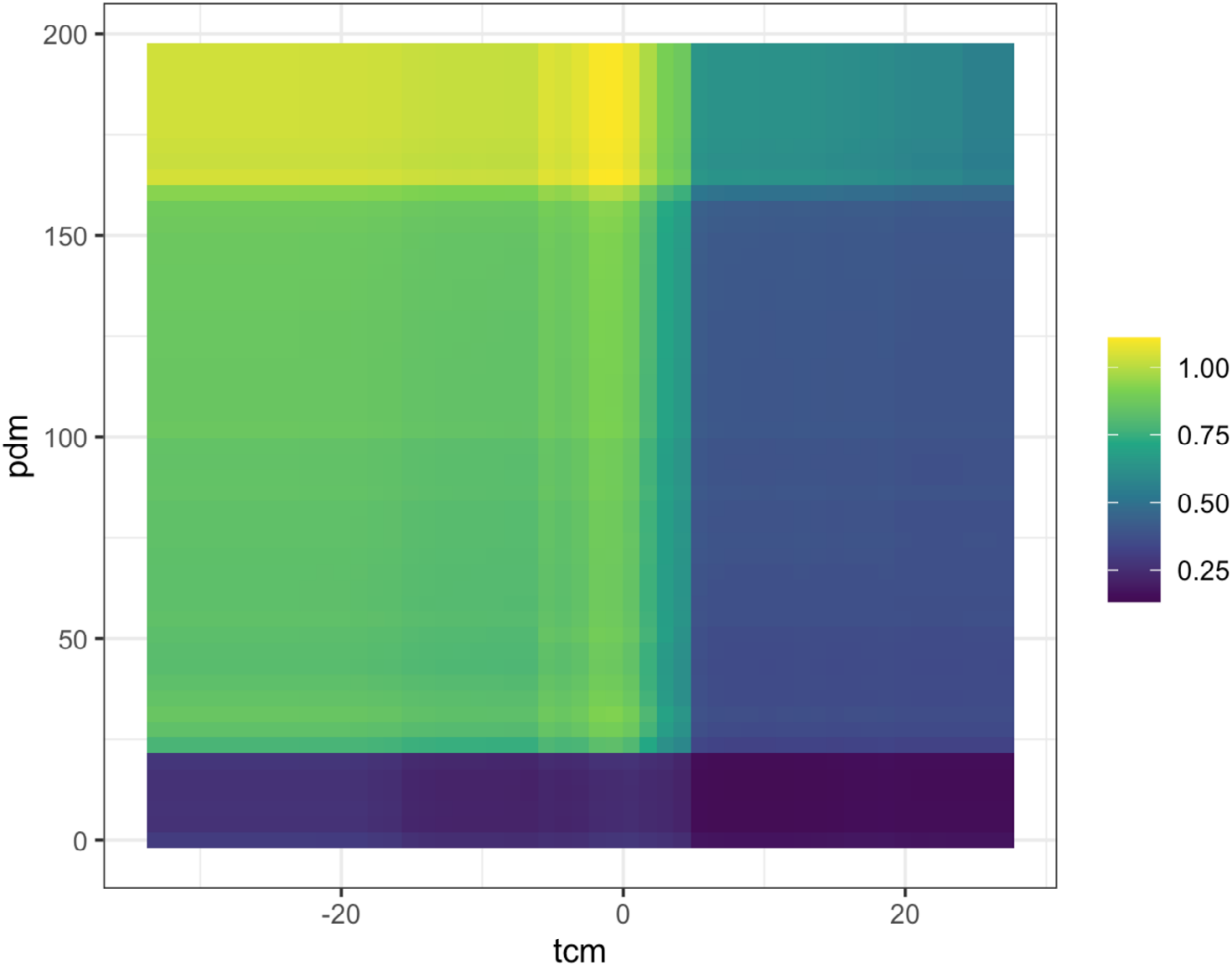
Partial dependence of the number of occurrence of *E. ferox* on the two climatic variables, temperature in the coldest month (tcm; °C) and precipitation in the driest month (pdm; mm), based on the random forest regression model. The blue‐to‐yellow gradient illustrates the estimated number of occurrence per grid by marginalizing over the effects of the other 11 explanatory variables.

The importance of the 13 variables measured by %IncMSE (Figure 5a) and by IncNodePurity (Figure 5b) varies with consistency in the most important ones as the human activity variables (noy for %IncMSE and GDP for IncNodePurity) and the second most important as the climate variable (tcm). We present partial dependence of the number of occurrences on the five most important variables based on both criteria (noy, ai, and hydlup in Figure 6; pdm and tcm in Figure 7), and report the partial dependence on the remaining 8 explanatory variables in Figure S2.

The partial dependence of the number of occurrence of *E. ferox* on the most important variable of human activity, that is, noy, indicates that its occurrence is strongly positively associated with anthropogenic nitrogen deposition as NO_y_ (Figure 6a). Aridity (ai) is also positively associated with its occurrence (Figure 6b), while the species is less likely to occur in the areas with short upstream flow of the rivers (hydlup) (Figure 6c). Following NO_y_, aridity, and upstream flow length, the partial dependence of its occurrence on the two climatic variables, namely temperature in the coldest month (tcm) and precipitation in the driest month (pdm), shows that *E. ferox* is unlikely to occur in the areas with lower than 20 mm precipitation in the driest month or those areas with the temperature in the coldest month over 5°C (Figure 7). Meanwhile, the species is likely to distribute in the areas (estimated number of occurrence per grid over 1.00) where there are over 160 mm precipitation in the driest month and sufficient freezing days in the coldest month (Figure 7).

### 3.3 Projected Distribution

**FIGURE 8:**
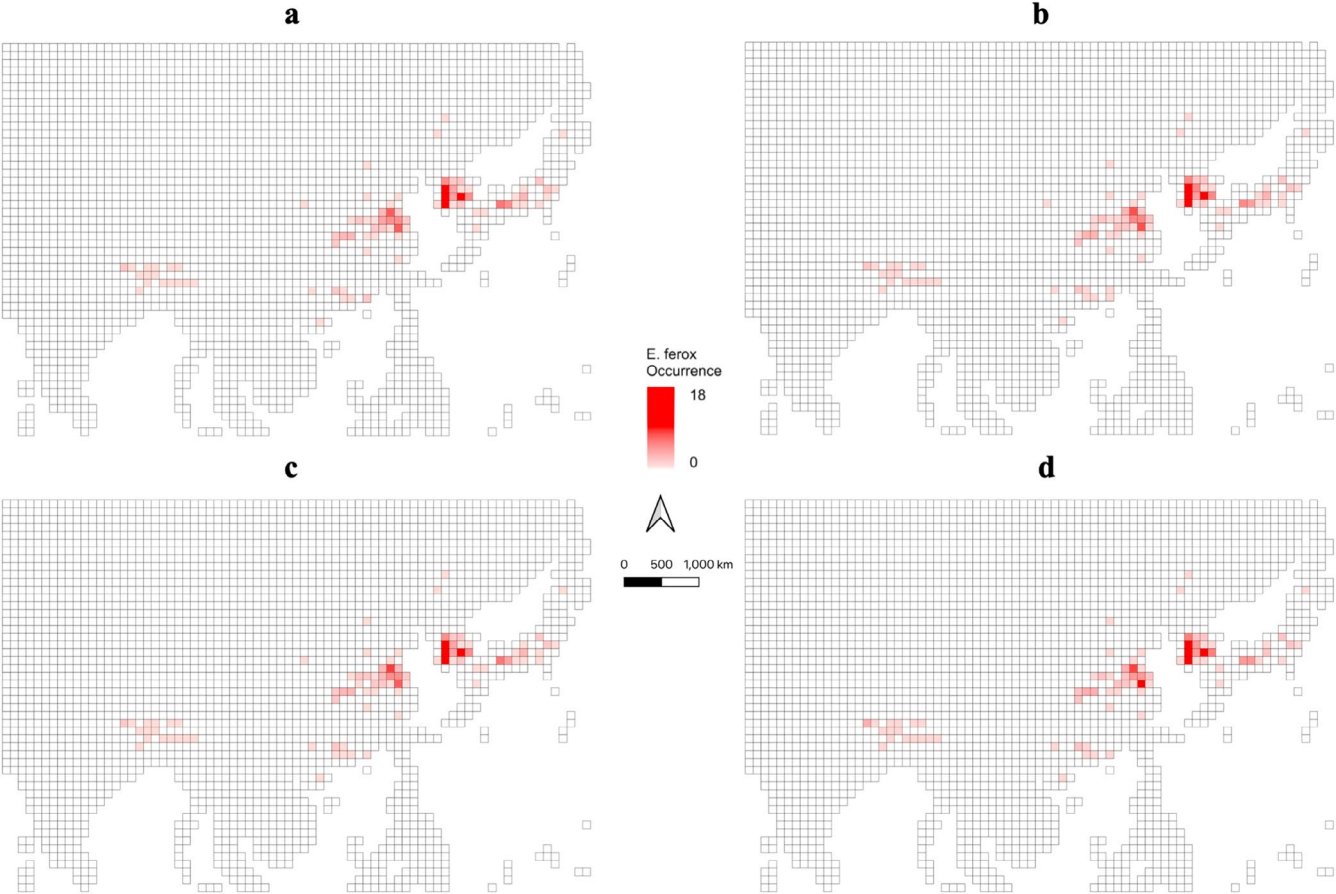
Projected number of occurrence of *E. ferox* per grid in the 2050s under the RCPs 2.6 (a), 4.5 (b), 6.0 (c), and 8.5 (d) climate change scenarios in the study area. The white‐to‐red gradient illustrates the number of occurrence per grid ranging from 0 to 18.

Based on temperature in the coldest month and precipitation in the driest month in the 2050s under four different climate change scenarios (RCPs 2.6, 4.5, 6.0, and 8.5), the projections for the numbers of occurrence of *E. ferox* per grid in the study area are shown in Figure 8. The distributions of the projected numbers of occurrence of the species under RCPs 2.6 (Figure 8a), 4.5 (Figure 8b), 6.0 (Figure 8c), and 8.5 (Figure 8d) are similar to the predicted current distribution (Figure 4b), all of which indicate the three identified clusters of hotspots of its observed distribution (Figure 3). However, the numbers of grids with at least one occurrence of the species decrease from the predicted (96) to the projected (86, 88, 88, and 80) under RCPs 2.6, 4.5, 6.0, and 8.5 in the 2050s, indicating 10.4%, 8.3%, 8.3%, and 16.7% decline in the area of its potential distribution, respectively. In terms of the changes in the numbers of occurrence from the present to the projected distribution of *E. ferox* under RCPs 2.6, 4.5, 6.0, and 8.5 scenarios in the 2050s, the means are −0.026, −0.028, −0.025, and −0.032, with the 1% quantiles as −0.77, −0.82, −0.70, and −0.99 and the 99% quantiles as 0.16, 0.19, 0.16, and 0.18, respectively, indicating potential decline in its distribution across the continent if other variables remain unchanged.

## 4 Discussion

Based on the by far most complete set of records of occurrence of *E. ferox* in Asia (*n* = 404; Figure 2), this study for the first time identifies the potential factors, not only climate and hydrogeography but also human activity, affecting its distribution which is clustered in north India, east China, and South Korea (Figure 3). Temperature in the coldest month plays the second most important role in predicting the distribution of *E. ferox* , which is negatively associated with the number of occurrences (Figure 7), a pattern also found in other Nymphaea species (Nzei et al. 2024). Apart from temperature, aridity index (Figure 6b) and precipitation in the driest month (Figure 7) are positively associated with its occurrence as expected given the physiological characteristics of *E. ferox* (Yang et al. 2020; Wu et al. 2022). When considering only the impact of climate, the expected number of occurrences of *E. ferox* decreases drastically at the temperature of the coldest month above 5°C (Figure 7). This might be due to the strong capability of cold stress tolerance (Yu et al. 2018) but weak tolerance of heat stress of the species (Zhang et al. 2020) with evidence from genetic analyses of Nymphaeales species including *E. ferox* (Zhang et al. 2020, 2026). Given that the rate of its genetic evolution is much slower than that of global warming (Quan et al. 2009; Imanishi et al. 2011; Kumar et al. 2016), the species is likely to suffer a rising extinction risk as the temperature of the coldest month increases above the optimal range of temperature for its growth and production. The situation might be more severe for its wild populations as competition for ecological niches by other macrophytes becomes stronger under climate change (Dalziell et al. 2019; Brykov et al. 2022; Liu et al. 2026). In fact, under mild to severe climate change scenarios, its distribution is projected to decline by 8.3%–16.7% in the 2050s with limited expansion of its potential habitats in Asia (Figure 8), such as in northwest China where its wild population went extinct by the end of the 20th century (Li et al. 2006). It is worth noting that the projected decline should be interpreted with caution as strength of human activity is projected to increase in India, China, and South Korea (Liu et al. 2020; Yang et al. 2022).

Over the long history of cultivation and utilization of the species in south and east Asia (Devi and Deka 2022; Zhang et al. 2022), it is not surprising that human activity, such as GDP and land use change to urban area, is more important than climate and hydrogeography for explaining the distribution of *E. ferox* (Figure 5). Specifically, the model predicts that the species is more likely to distribute in the areas with the majority of urban area than cropland, forest, and grassland (Figure S2b), corresponding to the three clusters of its occurrence in north India, east China, and South Korea (Figure 3). Anthropogenic nitrogen deposition as NO_y_ from the atmosphere to the surface is positively associated with the number of occurrences of the species (Figure 6a), as is the decadal percentage of land use change to urban area (Figure S2c). As a species of Nymphaeales, *E. ferox* is capable of growing and reproducing in eutrophic water contaminated with nitrogen, implying the potential promotion by anthropogenic nitrogen deposition from the atmosphere to the aquatic environment on its growth and reproduction (Wang et al. 2022). As a macrophyte, it absorbs common pollutants from residential wastewater (Rai et al. 2002; Ni et al. 2021; Brykov et al. 2022). These ecophysiological traits of *E. ferox* partly explain the positive association between human activity and its occurrence identified in this study. That being said, changes in human activity may contribute to the distribution of the species in Asia despite the limited expansion of its potential distribution under climate change (Figure 8) if conservation efforts pay off. However, although humans play important roles in cultivating it in south and east Asia (Devi and Deka 2022; Zhang et al. 2022), conservation for its wild populations is essential for maintaining its genetic diversity across Asia (Quan et al. 2009; Imanishi et al. 2011; Kumar et al. 2016), especially considering it being the only existing species of the genus *Euryale*.

The predicted current distribution and projections should be interpreted with caution as the random forest regression model explains only one fifth of variance in the number of occurrence per grid in the study area. Compared with the uncertainty of species distribution models for widely distributed plants (Chen et al. 2019), two major limitations in the model built for *E. ferox* are lack of water quality and other aquatic environment data and relatively limited distribution of the species in the continent. As a macrophyte sensitive to fluctuation in water quality (Imanishi and Imanishi 2014) and interspecific competition (National Institute of Biological Resources 2014), including variables of aquatic environment and ecology in the species distribution model would increase its accuracy, though currently these data are not available in most records. The other limitation is associated with the clustered distribution of the species in Asia (Figure 3), resulting in a large proportion of the grids without any record of occurrence and the zero‐inflation issue (Kalogirou 2016; Mushagalusa et al. 2022). Despite our best efforts for compiling and validating the records of the occurrence of *E. ferox* , there are additional records not included in this study, such as those in the Global Biodiversity Information Facility (GBIF). Considering the known issues of incomplete records for many plants in China (Qian et al. 2018), and because the records from GBIF are either duplicated or not in Asia, we did not include this data source. Our records are more complete than those from GBIF in the way that we compiled over 100 records of occurrence from 95 reports and news from the official channels of local, provincial and central government of China (Figure 1). To avoid overrepresentation of these records, we use 1‐degree grids to divide the study area and model the number of occurrence among these grids instead of analyzing the point pattern (Tan et al. 2025; Zhao et al. 2025). Apart from the data limitation, using statistical models such as geographically weighted zero‐inflated Poisson regression (GWZIPR) models (Kalogirou 2016), might be useful to predict the distribution of *E. ferox* . But the distribution of this species is highly clustered in three areas and the relationships between the occurrence and explanatory variables are far from linear (Figures 6 and 7; Figure S1), so we adopted random forest regression models, which are commonly used for species distribution modeling (Tan et al. 2025; Xu et al. 2025), in this study. Beyond random forest regression, supervised learning, such as neutral network, might enhance the performance of the machine learning‐based species distribution model if there were more records of occurrence available for the species (Guo et al. 2025). The spatial resolution of the grids in this study is 1‐degree, where local variation in environment and habitat conditions might not be captured. However, because the ratio between the grids with at least one record of occurrence of *E. ferox* and those without any record is already as small as 5.0%, dividing the grids into finer scales would lead to severe zero‐inflation and decrease model performance (Mushagalusa et al. 2022). Moreover, despite the efforts for ensuring completeness of occurrence data and prediction accuracy of the model, field investigation in its potential habitats, such as Li et al. (2006), should be conducted to validate the prediction.

## 5 Conclusion

This study for the first time finds that human activity plays an important role in predicting the distribution of the endangered aquatic plant, *Euryale ferox* , in Asia. Anthropogenic nitrogen deposition as NO_y_ positively associates with its occurrence while its distribution in Asia is projected to shrink under either mild, moderate, or severe climate change scenarios in the 2050s if human activity and habitat condition remain unchanged. Under the same condition, the potential habitats of the species are not likely to expand in Asia given that temperature in the coldest month is identified as a potential limiting factor for its distribution. With increasing interests in the economic and medical values of *E. ferox* in China and India, this study lightens the need to maintain sustainable cultivation and long‐term conservation for this species not only in its existing but also potential habitats in Asia.

## Author Contributions

**Yanxuan Li:** data curation (equal), formal analysis (equal), investigation (equal), methodology (equal), validation (equal), writing – original draft (equal). **Ningjing Zhang:** data curation (equal), investigation (equal), methodology (equal), validation (equal), writing – original draft (equal). **Zhongyi Gao:** data curation (equal), formal analysis (equal), investigation (equal), validation (equal), writing – original draft (equal). **Jiani Shi:** data curation (equal), investigation (equal), methodology (equal), visualization (equal), writing – original draft (equal). **Bingru Fang:** data curation (equal), formal analysis (equal), investigation (equal), software (equal), visualization (equal), writing – original draft (equal). **Jishu Guo:** methodology (equal), project administration (equal), resources (equal), software (equal), writing – original draft (equal). **Peng Xie:** funding acquisition (equal), resources (equal), supervision (equal), validation (equal), writing – original draft (equal). **Yun Zhang:** funding acquisition (equal), investigation (equal), project administration (equal), resources (equal), supervision (equal), validation (equal), writing – original draft (equal). **Wen Xiong:** investigation (equal), resources (equal), validation (equal), writing – original draft (equal). **Kun Xu:** conceptualization (equal), formal analysis (equal), funding acquisition (equal), methodology (equal), supervision (equal), writing – original draft (equal), writing – review and editing (equal).

## Funding

This study is funded by the Natural Science Foundation of Hubei Province (No: 2026AFB504, 2026AFC0029, and 2026AFC0031) by the Department of Science and Technology of Hubei Province, Project of Hubei Key Laboratory of Regional Development and Environmental Response (No: 2024(B)003) by Hubei University, and the National College Students Innovation and Entrepreneurship Training Program of Hubei Province (No: S202610513051) by Hubei Normal University.

## Conflicts of Interest

The authors declare no conflicts of interest.

## Supporting information


**Figure S1:** Correlation between the number of occurrence of *E. ferox* per grid and the 13 explanatory variables, except for the majority land use type in 2015 as a categorical variable, of the grids. The histograms of these variables are on the diagonal. Bottom left are the scatterplots between each pair of the variables. Top right panels show the Pearson correlation coefficient between each pair.
**Figure S2:** Partial dependence of the number of occurrence of *E. ferox* on topsoil pH (a), majority land use type in 2015 (b), percentage of land use change to urban area from 2005 to 2015 (c), elevation (d), topsoil organic carbon (e), soil and sedimentary deposit thickness (f), GDP (g), and decadal change in NDVI from 2005 to 2015 (h) based on the random forest regression model. The y axis is the estimated number of occurrence per grid by marginalizing over the effects of the other variables.

## Data Availability

The occurrence data are available at Zenodo (https://doi.org/10.5281/zenodo.18759570).

## References

[ece373994-bib-0001] Breiman, L. 2001. “Random Forests.” Machine Learning 45, no. 1: 5–32. 10.1023/A:1010933404324.

[ece373994-bib-0002] Brykov, V. , О. V. Polischuk , О. P. Bilous , V. А. Zhezherya , R. Brykova , and A. Neaman . 2022. “Photosynthetic Apparatus Features of *Nuphar lutea* and *Nymphaea alba* Floating Leaves Can Affect Their Redistribution.” Flora 292: 152080. 10.1016/j.flora.2022.152080.

[ece373994-bib-0003] Chen, X. , N. B. Dimitrov , and L. A. Meyers . 2019. “Uncertainty Analysis of Species Distribution Models.” PLoS One 14, no. 5: e0214190. 10.1371/journal.pone.0214190. 31120909 PMC6533036

[ece373994-bib-0004] Cui, D. , S. Liang , D. Wang , and Z. Liu . 2021. “A 1‐Km Global Dataset of Historical (1979–2017) and Future (2020–2100) Köppen‐Geiger Climate Classification and Bioclimatic Variables.” Earth System Science Data 13: 5087–5114. 10.5194/essd-13-5087-2021.

[ece373994-bib-0005] Dalziell, E. L. , C. C. Baskin , J. M. Baskin , R. E. Young , K. W. Dixon , and D. J. Merritt . 2019. “Morphophysiological Dormancy in the Basal Angiosperm Order Nymphaeales.” Annals of Botany 123, no. 1: 95–106. 10.1093/aob/mcy142. 30052753 PMC6344092

[ece373994-bib-0006] Das, S. , P. Der , U. Raychaudhuri , N. Maulik , and D. K. Das . 2006. “The Effect of *Euryale ferox* (Makhana), an Herb of Aquatic Origin, on Myocardial Ischemic Reperfusion Injury.” Molecular and Cellular Biochemistry 289, no. 1: 55–63. 10.1007/s11010-006-9147-1. 16628469

[ece373994-bib-0007] Devi, M. B. , and S. C. Deka . 2022. “Physicochemical Properties and Structure of Starches of Foxnut ( *Euryale ferox* Salisb.) From India and Its Application.” Journal of Food Processing and Preservation 46, no. 2: e16262. 10.1111/jfpp.16262.

[ece373994-bib-0008] Doxsey‐Whitfield, E. , K. MacManus , S. B. Adamo , et al. 2015. “Taking Advantage of the Improved Availability of Census Data: A First Look at the Gridded Population of the World, Version 4.” Papers in Applied Geography 1, no. 3: 226–234. 10.1080/23754931.2015.1014272.

[ece373994-bib-0009] Dutilleul, P. , P. Clifford , S. Richardson , and D. Hemon . 1993. “Modifying the t Test for Assessing the Correlation Between Two Spatial Processes.” Biometrics 49, no. 1: 305–314. 10.2307/2532625. 2720048

[ece373994-bib-0010] Evans, J. S. , M. A. Murphy , Z. A. Holden , and S. A. Cushman . 2011. “Modeling Species Distribution and Change Using Random Forest.” In Predictive Species and Habitat Modeling in Landscape Ecology, edited by C. Drew , Y. Wiersma , and F. Huettmann , 139–159. Springer. 10.1007/978-1-4419-7390-0_8.

[ece373994-bib-0011] FAO , and IIASA . 2023. Harmonized World Soil Database, Version 2.0, 69. Food and Agriculture Organization of the United Nation. 10.4060/cc3823en.

[ece373994-bib-0012] Farooqi, R. , H. Maryam , H. M. Jaffar , and T. Malik . 2025. “Therapeutic Potential of Fox Nut ( *Euryale ferox* ) in Osteoarthritis Management: A Nutraceutical Approach.” Food Reviews International 2025: 2541850. 10.1080/87559129.2025.2541850.

[ece373994-bib-0013] Getis, A. , and J. K. Ord . 1992. “The Analysis of Spatial Association by Use of Distance Statistics.” Geographical Analysis 24, no. 3: 189–206. 10.1111/j.1538-4632.1992.tb00261.x.

[ece373994-bib-0014] Greenwell, B. M. 2017. “pdp: An R Package for Constructing Partial Dependence Plots.” R Journal 9, no. 1: 421–436. 10.32614/rj-2017-016.

[ece373994-bib-0015] Guo, J. , Y. Huang , and Y. Zhang . 2025. “Supervised Learning‐Based Water Quality Prediction and Ecological Risk Factor Mining Across China's 12 Major River Basins.” Environmental Modelling & Software 197: 106840. 10.1016/j.envsoft.2025.106840.

[ece373994-bib-0016] Guru, P. N. , D. Mridula , R. K. Vishwakarma , and M. Gupta . 2024. “Lesser Grain Borer, *Rhyzopertha dominica* Infests Speciality Food Crop Makhana, *Euryale ferox* in India.” International Journal of Pest Management 2024: 2334229. 10.1080/09670874.2024.2334229.

[ece373994-bib-0017] Han, W. J. , J. Y. Cao , J. L. Liu , J. Jiang , and J. Ni . 2019. “Impacts of Nitrogen Deposition on Terrestrial Plant Diversity: A Meta‐Analysis in China.” Journal of Plant Ecology 12, no. 6: 1025–1033. 10.1093/jpe/rtz036.

[ece373994-bib-0018] Hastings, D. A. , P. K. Dunbar , and A. M. Hittelman . 2000. “Assessing the Global Land One‐Km Base Elsevation DEM.” In Geodesy Beyond 2000, edited by K. P. Schwarz , vol. 121. International Association of Geodesy Symposia. 10.1007/978-3-642-59742-8_16.

[ece373994-bib-0019] Huang, Y. , X. Ji , T. Su , et al. 2015. “Fossil Seeds of *Euryale* (Nymphaeaceae) Indicate a Lake or Swamp Environment in the Late Miocene Zhaotong Basin of Southwestern China.” Science Bulletin 60, no. 20: 1768–1777. 10.1007/s11434-015-0870-4.

[ece373994-bib-0020] Huo, Z. , X. Dai , S. Feng , S. Kang , and G. Huang . 2013. “Effect of Climate Change on Reference Evapotranspiration and Aridity Index in Arid Region of China.” Journal of Hydrology 492: 24–34. 10.1016/j.jhydrol.2013.04.011.

[ece373994-bib-0021] Imanishi, A. , and J. Imanishi . 2014. “Seed Dormancy and Germination Traits of an Endangered Aquatic Plant Species, *Euryale ferox* Salisb. (Nymphaeaceae).” Aquatic Botany 119: 80–83. 10.1016/j.aquabot.2014.08.001.

[ece373994-bib-0022] Imanishi, A. , S. Kaneko , Y. Isagi , J. Imanishi , Y. Natuhara , and Y. Morimoto . 2011. “Development of Microsatellite Markers for *Euryale ferox* (Nymphaeaceae), an Endangered Aquatic Plant Species in Japan.” American Journal of Botany 98, no. 8: e233–e235. 10.3732/ajb.1100056. 21821589

[ece373994-bib-0023] Jha, V. , A. N. Kargupta , R. N. Dutta , U. N. Jha , R. K. Mishra , and K. C. Saraswati . 1991. “Utilization and Conservation of *Euryale ferox* Salisbury in Mathila (North Bihar), India.” Aquatic Botany 39, no. 3–4: 295–314. 10.1016/0304-3770(91)90005-P.

[ece373994-bib-0024] Jiang, J. , H. Ou , R. Chen , H. Lu , L. Zhou , and Z. Yang . 2023. “The Ethnopharmacological, Phytochemical, and Pharmacological Review of *Euryale ferox* Salisb.: A Chinese Medicine Food Homology.” Molecules 28, no. 11: 4399. 10.3390/molecules28114399. 37298878 PMC10254204

[ece373994-bib-0025] Jiang, T. , T. Mou , F. Wang , et al. 2025. “The Impact of Global Change on the Slow Decline in Human‐Wild Boar ( *Sus scrofa* ) Conflicts in Central China From 2018 to 2022.” Global Ecology and Conservation 62: e03790. 10.1016/j.gecco.2025.e03790.

[ece373994-bib-0026] Kadono, Y. , and E. L. Schneider . 1987. “The Life History of *Euryale ferox* Salisb. in Southwestern Japan With Special Reference to Reproductive Ecology.” Plant Species Biology 2, no. 1–2: 109–115. 10.1111/j.1442-1984.1987.tb00037.x.

[ece373994-bib-0027] Kalogirou, S. 2016. “Destination Choice of Athenians: An Application of Geographically Weighted Versions of Standard and Zero Inflated Poisson Spatial Interaction Models.” Geographical Analysis 48, no. 2: 191–230. 10.1111/gean.12092.

[ece373994-bib-0028] Kapoor, S. , A. Kaur , R. Kaur , V. Kumar , and M. Choudhary . 2022. “ *Euryale ferox* , a Prominent Superfood: Nutritional, Pharmaceutical, and Its Economical Importance.” Journal of Food Biochemistry 46, no. 12: e14435. 10.1111/jfbc.14435. 36183158

[ece373994-bib-0029] Kashyap, A. , and O. J. Shukla . 2024. “A SD Approach for Optimizing Food Supply Chain Cost: A Case of Foxnut (Makhana) Industry.” In Responsible Production and Consumption, edited by P. K. Mishra , S. Prasad , B. Kamaiah , B. R. Shekhar , C. Behera , and P. K. Jena , 77–82. CRC Press. 10.1201/9781003542506-10.

[ece373994-bib-0030] Kim, J. Y. , G. Y. Kim , Y. Do , H. S. Park , and G. J. Joo . 2018. “Relative Importance of Hydrological Variables in Predicting the Habitat Suitability of *Euryale ferox* Salisb.” Journal of Plant Ecology 11, no. 2: 169–179. 10.1093/jpe/rtw106.

[ece373994-bib-0031] Kim, S. S. , Y. S. Kim , S. G. Ha , and H. T. Shin . 2010. “Dispersion of Vascular Plant in Daepyeong Swamp and Jilnal Swamp, Korea.” Journal of Korean Nature 3, no. 3: 187–198. 10.1016/S1976-8648(14)60023-2.

[ece373994-bib-0032] Kumar, H. , P. Priya , N. Singh , et al. 2016. “RAPD and ISSR Marker‐Based Comparative Evaluation of Genetic Diversity Among Indian Germplasms of *Euryale ferox* : An Aquatic Food Plant.” Applied Biochemistry and Biotechnology 180, no. 7: 1345–1360. 10.1007/s12010-016-2171-z. 27364330

[ece373994-bib-0033] Kummu, M. , M. Kosonen , and S. Masoumzadeh Sayyar . 2025. “Downscaled Gridded Global Dataset for Gross Domestic Product (GDP) Per Capita PPP Over 1990–2022.” Scientific Data 12, no. 1: 178. 10.1038/s41597-025-04487-x. 39885148 PMC11782586

[ece373994-bib-0034] Lee, S. E. , E. M. Ju , and J. H. Kim . 2002. “Antioxidant Activity of Extracts From *Euryale ferox* Seed.” Experimental & Molecular Medicine 34, no. 2: 100–106. 10.1038/emm.2002.15. 12085984

[ece373994-bib-0035] Lee, T. Y. , and W. Y. Liu . 2026. “Case in Taiwan Demonstrates How Corporate Demand Converts Payments for Ecosystem Services Into Long‐Run Incentives.” Agriculture 16, no. 2: 224. 10.3390/agriculture16020224.

[ece373994-bib-0036] Lehner, B. , K. Verdin , and A. Jarvis . 2008. “New Global Hydrography Derived From Spaceborne Elevation Data.” Eos, Transactions American Geophysical Union 89, no. 10: 93–94. 10.1029/2008eo100001.

[ece373994-bib-0037] Li, M. , S. Cao , Z. Zhu , Z. Wang , R. B. Myneni , and S. Piao . 2023. “Spatiotemporally Consistent Global Dataset of the GIMMS Normalized Difference Vegetation Index (PKU GIMMS NDVI) From 1982 to 2022.” Earth System Science Data 15, no. 9: 4181–4203. 10.5194/essd-15-4181-2023.

[ece373994-bib-0038] Li, X. X. , B. Liu , L. Wang , et al. 2026. “Impact of Climate Change and Human Activities on Suitable Distribution of *Rhodiola* Species in the Qinghai‐Tibet Plateau: Modeling Insights for Conservation Prioritization.” Ecology and Evolution 16, no. 1: e72896. 10.1002/ece3.72896. 41509563 PMC12778409

[ece373994-bib-0039] Li, Z. , D. Yu , W. Xiong , D. Wang , and M. Tu . 2006. “Aquatic Plants Diversity in Arid Zones of Northwest China: Patterns, Threats and Conservation.” Biodiversity and Conservation 15, no. 11: 3417–3444. 10.1007/s10531-005-0769-5.

[ece373994-bib-0040] Liu, H. , P. Gong , J. Wang , N. Clinton , Y. Bai , and S. Liang . 2020. “Annual Dynamics of Global Land Cover and Its Long‐Term Changes From 1982 to 2015.” Earth System Science Data 12, no. 2: 1217–1243. 10.5194/essd-12-1217-2020.

[ece373994-bib-0041] Liu, X. , Z. He , Y. Yin , X. Xu , W. Wu , and L. Li . 2018. “Transcriptome Sequencing and Analysis During Seed Growth and Development in *Euryale ferox* Salisb.” BMC Genomics 19, no. 1: 343. 10.1186/s12864-018-4707-9. 29743016 PMC5944168

[ece373994-bib-0042] Liu, X. , Y. Zhang , W. Han , et al. 2013. “Enhanced Nitrogen Deposition Over China.” Nature 494, no. 7438: 459–462. 10.1038/nature11917. 23426264

[ece373994-bib-0043] Liu, Y. , C. Mangoi , Z. Dong , et al. 2026. “Heterophyllous Plants Reorganize Plant Trait Coordination Between Floating and Emergent Habitats.” Plant Physiology and Biochemistry 234: 111364. 10.1016/j.plaphy.2026.111364. 42102489

[ece373994-bib-0044] Löhne, C. , M. J. Yoo , T. Borsch , et al. 2008. “Biogeography of Nymphaeales: Extant Patterns and Historical Events.” Taxon 57, no. 4: 1123‐19E. 10.1002/tax.574008.

[ece373994-bib-0045] Manish, K. 2022. “Medicinal Plants in Peril due to Climate Change in the Himalaya.” Ecological Informatics 68: 101546. 10.1016/j.ecoinf.2021.101546.

[ece373994-bib-0046] Mir, A. H. , S. Tyub , and A. N. Kamili . 2020. “Ecology, Distribution Mapping and Conservation Implications of Four Critically Endangered Endemic Plants of Kashmir Himalaya.” Saudi Journal of Biological Sciences 27, no. 9: 2380–2389. 10.1016/j.sjbs.2020.05.006. 32884420 PMC7451753

[ece373994-bib-0047] Moran, P. A. 1950. “Notes on Continuous Stochastic Phenomena.” Biometrika 37, no. 1/2: 17–23. 10.2307/2332142. 15420245

[ece373994-bib-0048] Mushagalusa, C. A. , A. B. Fandohan , and R. Glèlè Kakaï . 2022. “Random Forests in Count Data Modelling: An Analysis of the Influence of Data Features and Overdispersion on Regression Performance.” Journal of Probability and Statistics 2022, no. 1: 2833537. 10.1155/2022/2833537.

[ece373994-bib-0049] National Institute of Biological Resources . 2014. Korean Red List of Threatened Species, 2nd ed., 242. Ministry of Climate, Energy and Environment of the Republic of Korea. ISBN 9788968111037 93400.

[ece373994-bib-0050] Ni, M. , J. Yuan , L. Zhang , J. Hua , H. Rong , and Z. Gu . 2021. “In‐Situ and Ex‐Situ Purification Effect of Ecological Ponds of *Euryale ferox* Salisb on Shrimp Aquaculture.” Aquaculture 540: 736678. 10.1016/j.aquaculture.2021.736678.

[ece373994-bib-0051] Ning, J. , J. Liu , W. Kuang , et al. 2018. “Spatiotemporal Patterns and Characteristics of Land‐Use Change in China During 2010–2015.” Journal of Geographical Sciences 28, no. 5: 547–562. 10.1007/s11442-018-1490-0.

[ece373994-bib-0052] Nzei, J. M. , N. Martínez‐Médez , V. M. Mwanzia , et al. 2024. “Climatic Niche Evolution and Niche Conservatism of *Nymphaea* Species in Africa, South America, and Australia.” BMC Plant Biology 24, no. 1: 476. 10.1186/s12870-024-05141-1. 38816799 PMC11137912

[ece373994-bib-0053] Padala, V. K. , N. Ramya , M. Kumar , et al. 2023. “Spatial Distribution and Optimum Sample Size for Monitoring of Water Lily Aphid *Rhopalosiphum nymphaeae* (L.) in Makhana *Euryale ferox* Salisb.” International Journal of Tropical Insect Science 43, no. 6: 2167–2177. 10.1007/s42690-023-01119-y.

[ece373994-bib-0054] Page, M. J. , J. E. McKenzie , P. M. Bossuyt , et al. 2021. “The PRISMA 2020 Statement: An Updated Guideline for Reporting Systematic Reviews.” BMJ 372: n71. 10.1136/bmj.n71. 33782057 PMC8005924

[ece373994-bib-0055] Pelletier, J. D. , P. D. Broxton , P. Hazenberg , et al. 2016. “A Gridded Global Data Set of Soil, Intact Regolith, and Sedimentary Deposit Thicknesses for Regional and Global Land Surface Modeling.” Journal of Advances in Modeling Earth Systems 8, no. 1: 41–65. 10.1002/2015MS000526.

[ece373994-bib-0056] Qian, H. , T. Deng , J. Beck , et al. 2018. “Incomplete Species Lists Derived From Global and Regional Specimen‐Record Databases Affect Macroecological Analyses: A Case Study on the Vascular Plants of China.” Journal of Biogeography 45, no. 12: 2718–2729. 10.1111/jbi.13462.

[ece373994-bib-0057] Quan, Z. , L. Pan , W. Ke , and Y. Ding . 2009. “Polymorphic Microsatellite Markers in *Euryale ferox* Salisb.(Nymphaeaceae).” Molecular Ecology Resources 9, no. 1: 330–332. 10.1111/j.1755-0998.2008.02402.x. 21564641

[ece373994-bib-0058] Rai, U. N. , R. D. Tripathi , P. Vajpayee , V. Jha , and M. B. Ali . 2002. “Bioaccumulation of Toxic Metals (Cr, Cd, Pb and Cu) by Seeds of *Euryale ferox* Salisb. (Makhana).” Chemosphere 46, no. 2: 267–272. 10.1016/S0045-6535(01)00087-X. 11827284

[ece373994-bib-0059] Rao, S. , W. Qi , H. Cao , et al. 2026. “Faster Weight Growth in Invasive Mosquitofish *Gambusia holbrooki* and *Gambusia affinis* (Poeciliidae) Under Climate Change.” Ecology and Evolution 16, no. 1: e72943. 10.1002/ece3.72943. 41537139 PMC12796510

[ece373994-bib-0060] Riahi, K. , S. Rao , V. Krey , et al. 2011. “RCP 8.5—A Scenario of Comparatively High Greenhouse Gas Emissions.” Climatic Change 109, no. 1: 33–57. 10.1007/s10584-011-0149-y.

[ece373994-bib-0061] Runfola, D. , A. Anderson , H. Baier , et al. 2020. “geoBoundaries: A Global Database of Political Administrative Boundaries.” PLoS One 15, no. 4: e0231866. 10.1371/journal.pone.0231866. 32330167 PMC7182183

[ece373994-bib-0062] Semwal, D. P. , A. Pandey , P. G. Gore , S. P. Ahlawat , S. K. Yadav , and A. Kumar . 2021. “Habitat Prediction Mapping Using BioClim Model for Prioritizing Germplasm Collection and Conservation of an Aquatic Cash Crop ‘Makhana’( *Euryale ferox* Salisb.) in India.” Genetic Resources and Crop Evolution 68, no. 8: 3445–3456. 10.1007/s10722-021-01265-7.

[ece373994-bib-0063] Sinha, P. , A. E. Gaughan , F. R. Stevens , J. J. Nieves , A. Sorichetta , and A. J. Tatem . 2019. “Assessing the Spatial Sensitivity of a Random Forest Model: Application in Gridded Population Modeling.” Computers, Environment and Urban Systems 75: 132–145. 10.1016/j.compenvurbsys.2019.01.006.

[ece373994-bib-0064] Sokolova, M. , N. Japkowicz , and S. Szpakowicz . 2006. “Beyond Accuracy, F‐Score and ROC: A Family of Discriminant Measures for Performance Evaluation.” In Advances in Artificial Intelligence. AI 2006. Lecture Notes in Computer Science, edited by A. Sattar and B. Kang , vol. 4304. Springer. 10.1007/11941439_114.

[ece373994-bib-0065] Srivastava, J. N. , A. K. Singh , and N. Kotwal . 2022. “Disease and Pest Spectrum in Gorgon Nut ( *Euryale ferox* Salisbury) Crop and Management Strategy: Indian Scenario.” In Diseases of Horticultural Crops: Diagnosis and Management, edited by J. N. Srivastava and A. K. Singh , 227–250. Apple Academic Press. 10.1201/9781003160472-14.

[ece373994-bib-0066] Tan, M. , S. He , W. Shang , et al. 2025. “Human Activity Positively Associated With the Risk of Spread of the Giant African Snail (*Lissachatina fulica*) Recently Reported in Central and Northern China.” Global Ecology and Conservation 59: e03579. 10.1016/j.gecco.2025.e03579.

[ece373994-bib-0067] Tang, C. , W. Shang , M. Ji , et al. 2026. “Impacts of Topography and Canopy Height on Tree Diversity Patterns at a Major Montane Ecosystem in Central China.” Trees, Forests and People 26: 101340. 10.1016/j.tfp.2026.101340.

[ece373994-bib-0068] Thomson, A. M. , K. V. Calvin , S. J. Smith , et al. 2011. “RCP4.5: A Pathway for Stabilization of Radiative Forcing by 2100.” Climatic Change 109, no. 1: 77–94. 10.1007/s10584-011-0151-4.

[ece373994-bib-0069] Tian, H. , Z. Bian , H. Shi , et al. 2022. “History of Anthropogenic Nitrogen Inputs (HaNi) to the Terrestrial Biosphere: A 5 Arcmin Resolution Annual Dataset From 1860 to 2019.” Earth System Science Data 14: 4551–4568. 10.5194/essd-14-4551-2022.

[ece373994-bib-0070] Valavi, R. , J. Elith , J. J. Lahoz‐Monfort , and G. Guillera‐Arroita . 2021. “Modelling Species Presence‐Only Data With Random Forests.” Ecography 44, no. 12: 1731–1742. 10.1111/ecog.05615.

[ece373994-bib-0071] Wang, X. , A. Jain , B. Chen , et al. 2022. “Differential Efficacy of Water Lily Cultivars in Phytoremediation of Eutrophic Water Contaminated With Phosphorus and Nitrogen.” Plant Physiology and Biochemistry 171: 139–146. 10.1016/j.plaphy.2021.12.001. 34998101

[ece373994-bib-0072] Wu, P. , L. Zhang , K. Zhang , et al. 2022. “The Adaptive Evolution of *Euryale ferox* to the Aquatic Environment Through Paleo‐Hexaploidization.” Plant Journal 110, no. 3: 627–645. 10.1111/tpj.15717. PMC9314984 35218099

[ece373994-bib-0073] Xu, K. , J. Li , J. Zhang , D. Xing , and F. He . 2025. “How Many Trees Are There in the North American Boreal Forest?” Ecography 2025, no. 8: e07677. 10.1002/ecog.07677.

[ece373994-bib-0074] Yang, C. , H. Liu , Q. Li , et al. 2022. “Human Expansion Into Asian Highlands in the 21st Century and Its Effects.” Nature Communications 13, no. 1: 4955. 10.1038/s41467-022-32648-8. PMC9402921 36002452

[ece373994-bib-0075] Yang, Y. , P. Sun , L. Lv , et al. 2020. “Prickly Waterlily and Rigid Hornwort Genomes Shed Light on Early Angiosperm Evolution.” Nature Plants 6, no. 3: 215–222. 10.1038/s41477-020-0594-6. 32094642 PMC8075997

[ece373994-bib-0076] Yu, C. , G. Qiao , W. Qiu , et al. 2018. “Molecular Breeding of Water Lily: Engineering Cold Stress Tolerance Into Tropical Water Lily.” Horticulture Research 5: 73. 10.1038/s41438-018-0086-2. 30564371 PMC6265338

[ece373994-bib-0077] Zhang, J. , Y. Liang , G. Liu , et al. 2026. “Water Lily Complete Genomes Illuminate the Innovations of Water Lilies and Early Angiosperms.” Nature Plants 12: 937–953. 10.1038/s41477-026-02281-0. 42092166

[ece373994-bib-0078] Zhang, L. , Y. Chen , J. Zeng , et al. 2022. “Digestive and Physicochemical Properties of Small Granular Starch From *Euryale ferox* Seeds Growing in Yugan of China.” Food Biophysics 17, no. 1: 126–135. 10.1007/s11483-021-09706-7.

[ece373994-bib-0079] Zhang, L. , S. Wu , X. Chang , et al. 2020. “The Ancient Wave of Polyploidization Events in Flowering Plants and Their Facilitated Adaptation to Environmental Stress.” Plant, Cell & Environment 43, no. 12: 2847–2856. 10.1111/pce.13898. 33001478

[ece373994-bib-0080] Zhao, Y. , J. Liu , Q. Wang , et al. 2025. “Occurrence Data Sources Matter for Species Distribution Modeling: A Case Study of *Quercus variabilis* Based on Biomod2.” Ecology and Evolution 15, no. 5: e71390. 10.1002/ece3.71390. 40342697 PMC12061470

[ece373994-bib-0081] Zhou, J. , L. Zhou , and W. Xu . 2020. “Diversity of Wintering Waterbirds Enhanced by Restoring Aquatic Vegetation at Shengjin Lake, China.” Science of the Total Environment 737: 140190. 10.1016/j.scitotenv.2020.140190. 32783837

[ece373994-bib-0082] Zomer, R. J. , J. Xu , and A. Trabucco . 2022. “Version 3 of the Global Aridity Index and Potential Evapotranspiration Database.” Scientific Data 9, no. 1: 409. 10.1038/s41597-022-01493-1. 35840601 PMC9287331

