# Supplementary material for: Modeling Human Impact on the Distribution of the Only Existing Species of Euryale
Source: Ecol Evol. 2026 Jul 8;16(7):e73994. doi: 10.1002/ece3.73994 (PMC13345675; doi:10.1002/ece3.73994)
Supplement: Supplementary file 1 — Figure S1: Correlation between the number of occurrence of E. ferox per grid and the 13 explanatory variables, except for the majority land use type in 2015 as a categorical variable, of the grids. The histograms of these variables are on the diagonal. Bottom left are the scatterplots between each pair of the variables. Top right panels show the Pearson correlation coefficient between each pair. Figure S2: Partial dependence of the number of occurrence of E. ferox on topsoil pH (a), majority land use type in 2015 (b), percentage of land use change to urban area from 2005 to 2015 (c), elevation (d), topsoil organic carbon (e), soil and sedimentary deposit thickness (f), GDP (g), and decadal change in NDVI from 2005 to 2015 (h) based on the random forest regression model. The y axis is the estimated number of occurrence per grid by marginalizing over the effects of the other variables. [file ECE3-16-e73994-s001.docx]

**Supplementary Materials**


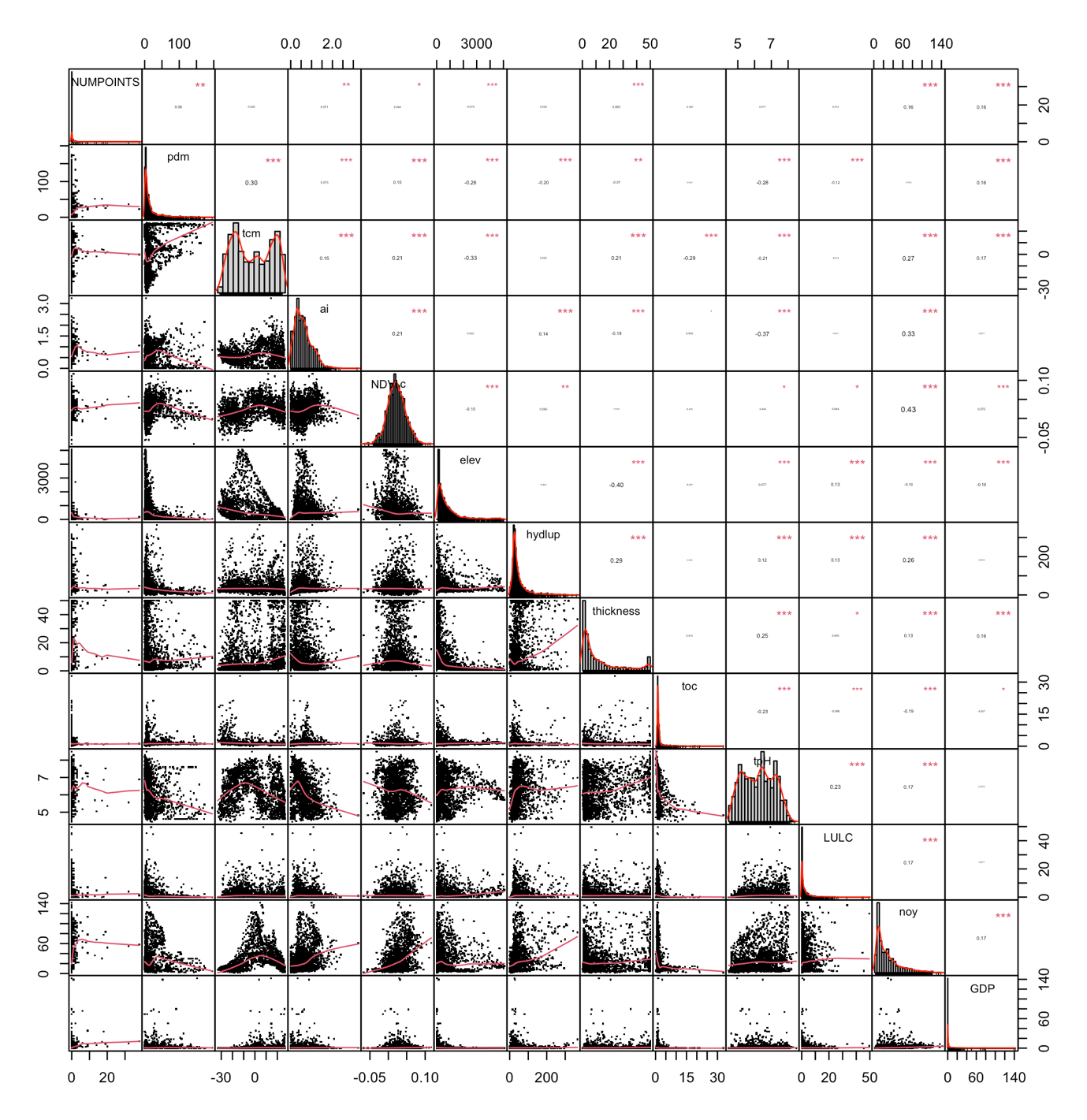


Figure S1 Correlation between the number of occurrence of *E. ferox* per grid and the 13 explanatory variables, except for the majority land use type in 2015 as a categorical variable, of the grids. The histograms of these variables are on the diagonal. Bottom left are the scatterplots between each pair of the variables. Top right panels show the Pearson correlation coefficient between each pair.


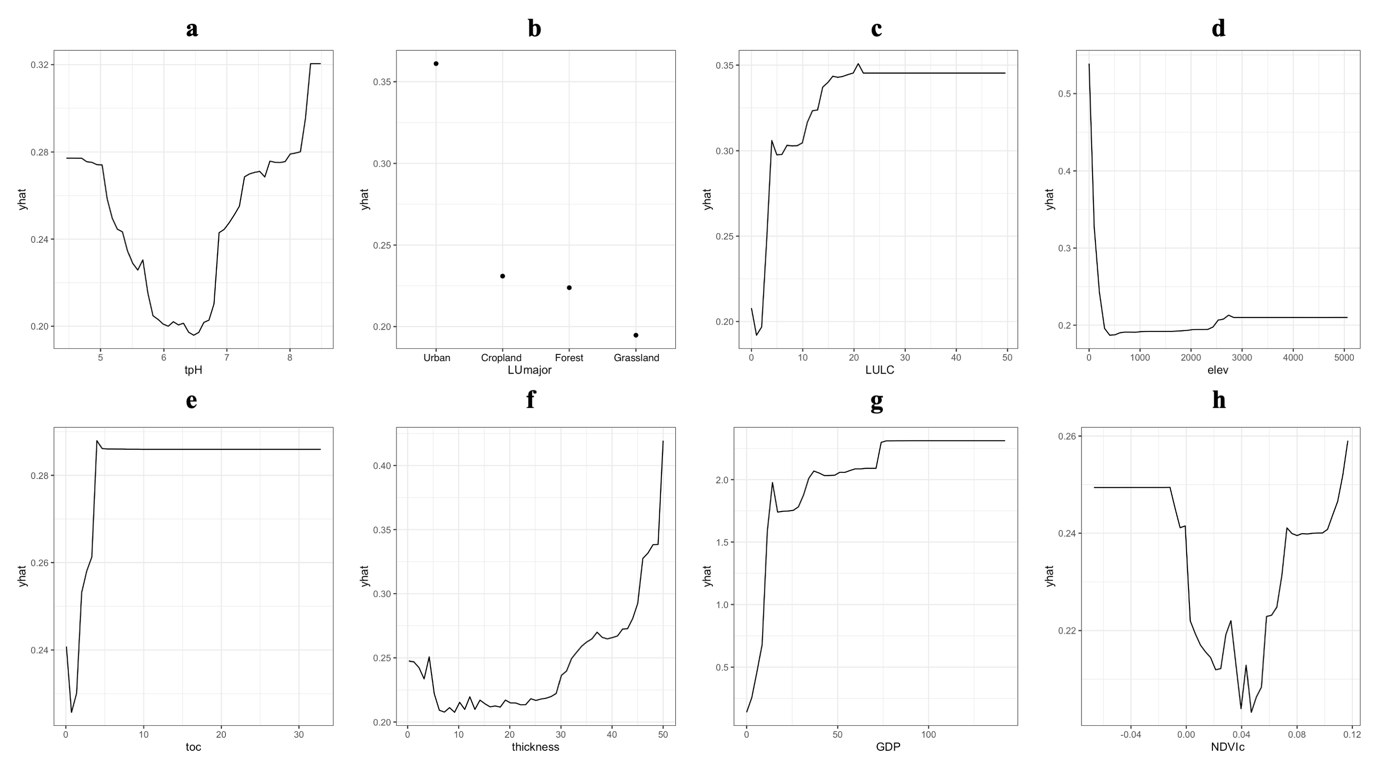


Figure S2 Partial dependence of the number of occurrence of *E. ferox* on topsoil pH (a), majority land use type in 2015 (b), percentage of land use change to urban area from 2005 to 2015 (c), elevation (d), topsoil organic carbon (e), soil and sedimentary deposit thickness (f), GDP (g), and decadal change in NDVI from 2005 to 2015 (h) based on the random forest regression model. The y axis is the estimated number of occurrence per grid by marginalizing over the effects of the other variables.
